# Adaptability to Online Teaching during Covid-19 Pandemic: A Multiple Mediation Analysis Based on Kolb’s Theory

**DOI:** 10.3390/ijerph18158032

**Published:** 2021-07-29

**Authors:** Camelia-Mădălina Răducu, Elena Stănculescu

**Affiliations:** Faculty of Psychology and Educational Sciences, University of Bucharest, 90 Panduri Street, 5th District, 030018 Bucharest, Romania

**Keywords:** distance education and online learning, teaching style, general self-efficacy, emotional intelligence, concrete experience learning style, teacher professional development

## Abstract

The process of transferring to online teaching during the pandemic COVID-19 lockdown has been a core issue for teachers around the globe. The main aim of this paper was to investigate the direct and indirect effects of emotional intelligence (EI) and general self-efficacy on the adaptability to online teaching (AOT). A multiple-mediation model was proposed, including the mediating effect via the teacher’s Facilitator role and teacher’s concrete experience learning mode (CE-LM), as defined in experiential learning theory (ELT). Methods: Data were collected from a sample of 330 preschool and primary school teachers (84 males, *M_age_* = 38.3, *SD* = 9.14). Path analysis was performed based on maximum likelihood estimation with the resampling method. Results: The findings showed that the proposed model fit the data well. A serial mediation path between EI and AOT via the teacher’s Facilitator role and CE-LM was obtained. In addition, CE-LM mediated the relationship between general self-efficacy and AOT. Conclusion: To date, this is the first study to analyse the direct and indirect effects of dispositional traits, such as EI and general self-efficacy, on AOT in the framework of Kolb’s ELT. Our results highlight the mediating mechanisms of this relationship, that is, the teacher’s Facilitator role and CE-LM. The current research provides an empirical body based on which new instructional strategies will be developed to improve the quality of online teaching during the COVID-19 pandemic and beyond.

## 1. Introduction

The coronavirus pandemic (COVID-19) has led to the closure of schools in countries from Europe 20 countries and the closure of preschools in 19 European countries and Central Asia. This has affected 49.8 million children, from preschoolers to high school students, who had a very disrupted last school semester, which culminated in the closure of schools [1]. In Romania, an estimated three million students from kindergarten to high school started learning online on 16 March 2020, until the end of the school year. In September, 80 percent of all students returned to their schools, but later, depending on the rate of COVID-19 infection locally, they switched back to online learning. However, there are still no concrete statistics related to the national practice of online teaching in the first semester of the 2020–2021 school year in the country.

## 2. Theoretical Background

### 2.1. AOT during the COVID-19 Pandemic

As the coronavirus pandemic spread, the need to rethink and redesign the instructional strategies increased to respond optimally to the rising demand for higher, continuing education. In this unprecedented situation, online education could reflect a pedagogical shift from the traditional method to the modern approach of teaching—learning [2]. Remote education could also contribute to democratisation and the evolution of the scholarship of teaching [3] by improving the ability to recognize which material is essential to the students’ understanding and learning, to logically and consistently organize and deliver course material, and to seek solutions to use all the benefits provided by new technologies in the instructional process [4]. Thus, adaptable and innovative teacher behavior could be considered central to a competitive society that is advancing technologically with astonishing speed by promoting innovation in all its aspects among students in schools [5].

However, in this context of benefits and challenges, teachers were forced to adapt in a very short time to online teaching. This process of adjustability has meant overcoming barriers, such as separation from their students [6], accessing technological and digital infrastructures, and rapidly developing the digital competences necessary for online teaching, as well as ensuring that pupils had access to online teaching and learning activities [7]. In addition, teachers were required to ensure good collaboration with parents and careful monitoring of children’s/students’ behavior to identify and combat possible effects determined by the changing learning methods and social realities [8]. Thus, although this period represents also a great opportunity for innovation in education, most teachers felt ineffective, and dissatisfied with online learning [9], even though they reported spending extra time becoming accustomed to the online teaching environment [10]. At the same time, they struggled in their personal lives to cope with stress and depressive symptoms due to the pandemic context [11]. In this sense of personal and professional challenges, increased levels of mental, social, and technical stress were reported among the teachers [12].

Thus, as we saw in the first part of the section, although this period also offered great opportunities for expanding teaching skills in other learning environments, such as online, the existing literature on the impacts of COVID-19 on teaching has predominantly focused only on the difficulties faced by teachers during the process of transferring to online teaching [13]. Less attention has been paid to identifying and supporting the acquisition of teaching capacities that promote online teaching effectiveness. Because as we have previously noticed, most teachers have encountered various difficulties in adapting to online teaching, we consider it necessary to highlight those capacities that support online teaching, of which we consider adaptability to online teaching (AOT) to be essential for an effective teacher. Moreover, we consider that the identification of those factors that promote AOT could help teachers seek viable solutions to improve their instructional strategies in an online environment.

### 2.2. What Influences AOT?

In this section, we analyse the conceptualisation of adaptability to online teaching (AOT), as well as its predictors such as teaching/learning styles, as defined by Kolb et al. [14], as well as dispositional traits such as emotional intelligence (EI) and general self-efficacy. First, the definition used in this study for adaptability to online learning was proposed by Martin et al. [15], who described it as individuals’ capacity to constructively regulate psycho-behavioral functions in response to new, changing, and/or uncertain circumstances, conditions, and situations. Teachers’ adaptability has been portrayed as a tripartite model to assess teachers’ cognitive, behavioral, and emotional adaptability [16].

#### 2.2.1. Teaching Roles and Learning Styles in Kolb’s Theory

Previous studies have shown that, in the pandemic context, teachers must not only assume the role of transmitting knowledge but also play the role of ‘leader’ and ‘accompanier’ through effective guidance and communication [17], because, although there are many opportunities, applications, and automated digital environments in the world, none can replace a teacher [18]. Thus, the first factor that may influence AOT it is represented by the teacher’s role or style. In this regard, experiential learning theory (ELT) [14] provides a comprehensive conceptual framework through which teachers around the world can benefit from a viable learning stylel to support the process of adapting to online learning and to provide innovative design ideas for training activities, even in a remote environment.

The experiential educator is an essential concept grounded in ELT that defines the role of teacher in terms of teaching style. To support educators in applying experiential education concepts, Kolb, Kolb, Passarelli, and Sharma [14] projected a framework—the Educator Role Profile (KERP)—that defines four key experiential educator roles: Facilitator, Subject Expert, Standard Setter/Evaluator, and Coach. The Facilitator and Coach are learner focus styles, and the Expert and Evaluator are subject focus styles. Each role represents bridges strategies [14] between the four modes of learning: concrete experience (CE), reflective observation (RO), abstract conceptualisation (AC), and active experimentation (AE). ‘The unique ways individuals spiral through the learning cycle based on their preference for the four different learning styles’ [19] forms the learning styles. The concrete experience learning mode (CE-LM) represents learning by ‘touching and feeling’, reflective observation (RO-LM) utilises ‘watching and listening’, abstract conceptualisation (AC-LM) is based on ‘thinking’, and active experimentation (AE-LM) involves ‘learning by doing’ [20].

Concerning the link between teaching style and leaning style, some studies have shown that teachers tend to teach in the way they learn [21,22]. In this regard, Kolb [14] found an extremely significant relationship between an abstract learning style, subject orientation, and the roles of Expert and Evaluator, while teachers who learn in a concrete much prefer the role of Facilitator.

As we have seen in the first part of this section, the findings of previous studies argue that online education requires a flexible, creative, close-to-the-student teaching style, open to guiding students through a concrete learning process that transcends the screen and promotes intrinsic motivation to discover and to learn. In this sense, the Facilitator role, as described in ELT, seems to best ensure the necessary premises for a successful AOT, with the specific concrete experience learning style.

**Hypothesis** **1** **(H1).** *A positive relationship was expected between AOT on one hand and the CE-LM and Facilitator role on the other.*

#### 2.2.2. AOT and Dispositional Traits

Another factor that the literature has regularly discussed as a strong predictor of adaptability to change is emotional intelligence (EI) [23,24,25,26]. At the same time, EI promotes success in online learning [27], improves readiness for online learning [28], and prevents boredom in remote courses [29]. Moreover, this challenging pandemic period required teachers to have even greater emotional resources than usual [30], to focus on increasing teacher–student connectedness using facial expressions and body language [30,31], and to maintain a cheerful disposition for improving students’ mental and physical well-being [32,33]. Thus, considering these findings, we can assume that EI promotes AOT.

Another dispositional trait that could play an important role in successful online teaching adaptability is general self-efficacy, defined as the individual’s ability to use his or her own knowledge and skills to achieve the proposed goals [34]. High general self-efficacy has been proven to augment teachers’ computer self-efficacy [35], subjective well-being [34], and job satisfaction [36]. As a particular form of self-efficacy, teacher self-efficacy has been shown to have a significant impact over teacher’s choices of personal goals, perseverance in the face of adversity [37] and motivation to use technology in the instructional–educational process [38].

**Hypothesis** **2** **(H2).** *Considering these findings, a positive association was expected between AOT and dispositional traits such as EI and general self-efficacy.*

#### 2.2.3. Teaching-Learning Mode and Dispositional Traits in AOT

As we saw in the first part of the theoretical background, most studies conducted during the pandemic focused on teachers’ challenges, but only a few studies provided evidence of what a teacher should do to best adapt to online teaching and respond as well as possible to the educational needs of students. For example, a study conducted during the pandemic [39] showed most students trained from the Facilitator role produced the highest average values on their final exam, which improved students’ enjoyment of the course [40]. Another study [41] suggested that the teacher’s role should be relevant, effective, and close enough to students without losing sight of the native side effects of excessive screen time. From this finding, we can extract that the teacher should possess a high level of EI to be close to students and efficacious to produce relevant education. Teachers should teach using concrete experience, not overuse the screen, and adopt a warm and affirmative teaching style as a Facilitator. As described by Kolb [14], the role of Facilitator seems to best combine the richness of emotional resources, expressiveness, and openness to concrete experiences, with increased resilience, optimal management of negative emotions, and perseverance in achieving personal goals.

**Hypothesis** **3** **(H3).** *Considering the mentioned aspects, it is expected that EI, general self-efficacy, CE-LM, and a teacher’s Facilitator role will positively predict the AOT.*

**Figure 1:**
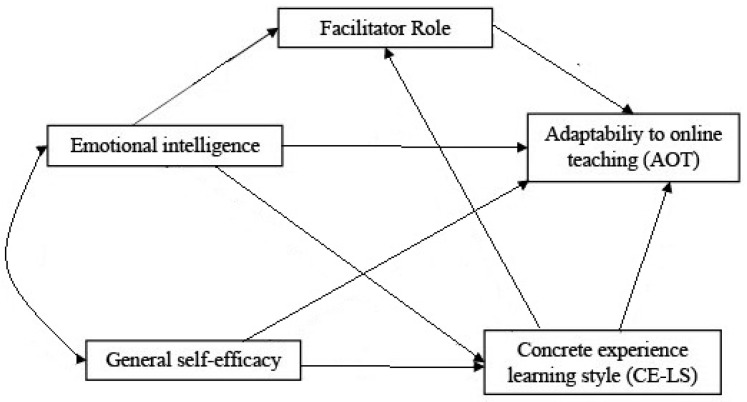
Proposed multiple mediation model.

Furthermore, a multiple mediation model demonstrating the relationship between five concepts—AOT, Facilitator Role, CE-LM, EI, and general self-efficacy—was proposed (Figure 1), and using the data collected from preschool and primary school teacher, the following hypothesis were tested:

**Hypothesis** **4** **(H4).** *The multiple-mediation model that includes the direct effect of EI and general self-efficacy of AOT, as well as indirect effects via the teacher’s Facilitator role, respectively, CE-LM, has a good fit with the data.*

**Hypothesis** **4a** **(H4a).** *The teacher’s facilitator role mediates the relationship between EI and AOT.*

**Hypothesis** **4b** **(H4b).** *CE-LM has a mediating role in the relationship between EI and AOT.*

**Hypothesis** **4c** **(H4c).** *Here, serial mediation between EI and AOT through CE-LM and the teacher’s Facilitator role.*

**Hypothesis** **4d** **(H4d).** *The relationship between general self-efficacy and AOT is mediated through CE-LM.*

## 3. Methods

### 3.1. Participants

The sample consisted of 330 (84 males, *M_age_* = 38.3, *SD* = 9.14) teachers from urban (65.8%) and rural (34.2%) settings, employed full time. Their reported teaching experience was less than one year (4.5%), between two and five years (10.9%), between five and 10 years (19.1%), between 10 and 20 years (25.5%), and more than 20 years (40%). The sample included preschool (*n* = 108) and primary school teachers (*n* = 222).

### 3.2. Procedure

The data were collected using an online survey via Google Forms. Participants were provided with an information sheet to read about the study and to consent before collecting data. The survey comprised two sections. The first section referred to participants’ demographic information, such as gender, teaching grades, years of teaching experience, and urban or rural teaching environment. The second section involved reporting the levels of adaptability, general self-efficacy, EI, and the preference for a certain type of educator role and learning style. The research was ethically conducted under the Helsinki Declaration 1975, as revised in 2000. Approval for the study was granted by the university ethics committee. The data were collected and processed, respecting all the rights and guarantees provided in EU Regulation 2016/679 and Organic Law 3/2018 of 5 December on the Protection of Personal Information and guarantee of digital rights. Data were collected with a snowball sampling technique carried out on social networks and instant messaging during spring break in April 2021. The link to the online survey was posted with a short description of its purpose, the length of time needed to complete it, and invitations for others to share the link. In exchange for completing the questionnaires, certificates of participation in the research were provided for the teacher’s personal file. The selection criteria for inclusion in this study were a primary or preschool level of teaching. All participants were voluntarily involved, with personal confidentiality guaranteed in all circumstances. They gave their written informed consent prior to filling out the questionnaire, after being informed of the research objectives and the anonymous nature of their answers.

### 3.3. Measures

The Adaptability to Online Teaching Scale (AOTS) [15] was adapted to the online teaching domain and has a two dimension structure: a behavior-cognitive factor (e.g., ‘I can revise the way I think about a new situation to help me through it’), and an affective factor (e.g., ‘I can minimise frustration or irritation so I can deal with it best’). This Likert scale ranged from 1—completely disagree to 7—strongly agree, which was first validated as a two-dimensional structure among high school students. In the present research, the Cronbach’s alpha for the entire scale was 0.94 (95% CI [0.93, 0.95]); 0.95 (95% CI [0.94, 0.96]) for the behavior—cognitive adaptability subscale; and 0.96 (95% CI [0.95, 0.97]) for the affective adaptability subscale, showing high internal consistency [42].

The Kolb Educator Role Profile (KERP) [14]. This self-assessment tool includes items for individual teaching styles, beliefs about teaching and learning, and goals for the educational process and instructional practices. KERP was ‘formatted in a forced-choice comparison series of 30 items’ [14], and each item relates to one of four educator roles: Facilitator (e.g., ‘I aim for learners to develop a lifelong love of learning’), Expert in the Subject Matter (e.g., ‘I share my subject matter knowledge and expertise’), Evaluator/Standard Setter (e.g., ‘I use tests to evaluate learners’ understanding of a subject’) and Coach (e.g., ‘I believe learning occurs best in a real-life context’). The pairing items are based on their statement type, and each role was paired to every other role three times. By adding the number of choices for each role, a score between zero and 15 is obtained. Combination scores were also calculated to determine a Subject Matter-versus Learner-centred ([Expert + Evaluator] − [Coach + Facilitator]) type of educator or an action versus meaning ([Evaluator + Coach] − [Facilitator + Expert]) focus of the educator. In the current study, the Cronbach’s alpha for the subscales were: Facilitator 0.83 (95% CI [0.82, 0.85]), Expert 0.63, 95% CI [0.62, 0.65]), Evaluator 0.57 (95% CI [0.56, 0.59]), and Coach 0.72 (95% CI [0.71, 0.74]).

The Kolb Learning Styles Inventory 4.0 (KLSI 4.0) [19] comprises 20 items (e.g., ‘I learn: Thinking/Watching/Doing/Feeling’), covering four primary scores that measure an individual’s relative emphasis on the four learning orientations—Concrete Experience (CE-LM), Reflective Observation (RO-LM), Abstract Conceptualisation (AC-LM), and Active Experimentation (AE-LM). Two combination scores measure an individual’s preference for abstractness over concreteness (AC-CE) and action over reflection (AE-RO) [19]. KLSI 4.0 describes 20 situations with four choices each. It has a four-point Likert-type scoring scheme where, for each situation, the most suitable choice is scored at four, the second-most suitable one is scored at three, the third-most suitable is scored 2, and the least suitable one is scored at one. In the present research, the Cronbach’s alpha for the subscales were: CE-LM 0.89 (95% CI [0.88, 0.91]), AC-LM 0.86 (95% CI [0.85, 0.87]), RO-LM 0.81 (95% CI [0.80, 0.83]), and AE-LM 0.79 (95% CI [0.78, 0.81]).

Trait Emotional Intelligence Questionnaire—Short Form for Adults (TEIQue-ASF); [43]. This scale consists of 30 items evaluated on a Likert scale from 1—completely disagree to 7—completely agree (e.g., ‘Expressing my emotions with words is not a problem for me’; ‘I often find it difficult to see things from another person’s viewpoint’). The global EIT score was calculated by inversely rating 15 of the 30 items. For the four subscales, the score was divided by the number of items in the scale. In the present research, Cronbach’s alpha was 0.90 (95% CI [0.88, 0.91]).

The General-Self Efficacy Scale (GSES) [44]. This psychometric scale has been used to measure the general sense of perceived self-efficacy and consists of 10 items (e.g., ‘If I try hard enough, I can usually handle whatever comes my way’) on a Likert scale from 1—not at all true to 4—exactly true. For the Romanian version of this scale, the Cronbach’s alpha coefficient was 0.78 [45]. In the present research, Cronbach’s alpha was 0.93 (95% CI [0.92, 0.94]).

### 3.4. Statistical Analysis

Statistical analyses used the Statistical Package for Social Sciences (SPSS 23) (IBM Corp. Released 2015. IBM SPSS Statistics for Windows, Armonk, NY, USA) and the Analysis of Moment Structure statistical package (AMOS 23) (IBM Corp. Released 2014., Chicago, IL, USA). First, descriptive statistics (mean, standard deviation) and skewness, kurtosis, univariate, and multivariate normality were computed. To test the first three hypotheses, Pearson product—moment correlations, and multiple linear regression analyses, were computed. The homoscedasticity of residuals in the regression and collinearity statistics were checked to ascertain the regression model was correct. Verifying the last hypothesis, a path analysis was conducted. Testing the statistical significance of the proposed theoretical model, three criteria suggested by Schumacker and Lomax [46] were considered: (i) a non-statistically significant chi-square test; (ii) the statistical significance of each parameter estimate; and (iii) the extent of the parameter estimates to show that they are consistent with the substantive theory. The estimation method was maximum likelihood (MLE) with the bootstrapping technique (with 5000 bootstrapped samples), as recommended by Byrne [47], for dealing with multivariate non-normal data. To calculate the total direct and indirect effects in the mediation model, the user-new estimands technique with the bias-corrected bootstrap method and 95% bootstrap confidence intervals was performed. Furthermore, several inferential goodness-of-fit statistics were used to determine the goodness of fit of the model. In addition to the chi-square test (χ2) and χ2/df, also used were the root mean square error of approximation (RMSEA), root mean square residual (RMR), standardised root means square residual (SRMR), goodness of fit index (GFI), adjusted goodness of fit index (AGFI), Bollen’s incremental fit index (IFI), Tucker-Lewis fit index (TLI), and Bentler comparative fit index (CFI). As Hu and Bentler [48] recommended, the cut-off criterion for GFI, AGFI, RFI, CFI, and TLI is ≥ 0.95, for RMSEA < 0.06, for RMR the smaller the better, with zero indicating perfect fit and for SRMR < 0.08.

## 4. Results

Checking the descriptive statistics, it was found that 19.1% of teachers had the highest score on EI (*n* = 63), 27% were characterised by high AOT (*n* = 89), and 18.2% obtained the highest scores on the facilitator role (60). More details on AOT depending on various socio-demographic variables can be seen in the Appendix A (Table A1). Descriptive statistics displayed in Table 1 showed that all the variables do not depart substantially from univariate normality, considering the cut-off criteria for skewness (<2) and kurtosis (<7), according to West et al. [49]. Mardia’s coefficient (6.132) and CR (3.213) proved the multivariate non-normality of the data.

Based on the Pearson’s product—moment correlation coefficients obtained (as shown in Table 2), the first two hypotheses were confirmed. As expected, AOT was positively associated with EI, general self-efficacy, CE-LM, and the Facilitator role. More specifically, we obtained a strong connection between AOT and EI, respectively, moderate associations between AOT on one hand and general self-efficacy, and CE-LM and the facilitator role on the other.

The third hypothesis presupposing the predictive role of EI, general self-efficacy, CE-LM, and the facilitator role on AOT was validated. The results of multiple regression analysis indicated that the four hypothesised predictors explained 28.7% of the variance for AOT [F (4325) = 86.42, *p* < 0.001, R2 = 0.51]. More precisely, we obtained that EI (b = 0.34, t (325) = 5.68, *p* < 0.001), general self-efficacy (b = 0.18, t (325) = 3.47, *p* < 0.001), CE-LM (b = 0.19, t (325) = 3.59, *p* < 0.001), and the Facilitator role (b = 0.16, t (325) = 2.70, *p* < 0.001) significantly predicted AOT. An examination of residual statistics provided evidence that the residuals’ mean was 0.00. As Field [50] recommended, this value indicates that the regression model was good at explaining the evolution of the criterion. Additionally, collinearity statistics proved the independence of the residuals. More precisely, the lowest VIF coefficient was 1.90, and the highest, 2.53. The lowest tolerance coefficient was 0.39, and the highest was 0.52. A normal P-P plot of regression standardised residuals and rectangular scatterplot with standardised residual values between −3 and 3 highlighted the homoscedasticity or normal distribution of the residuals.

The absolute fit index (χ2 = 1.74, df = 1) and non-significant *p*-value (*p* = 0.186) illuminated a good fit to the data, with GFI = 0.99, AGFI = 0.96, CFI = 0.99, RFI = 0.97, TLI = 0.99, RMSEA = 0.04 (90% CI [0.00, 0.16]), RMR = 0.02, and SRMR = 0.01. All these results supplied evidence for the excellent fit of the theoretical model to the data.

**Figure 2:**
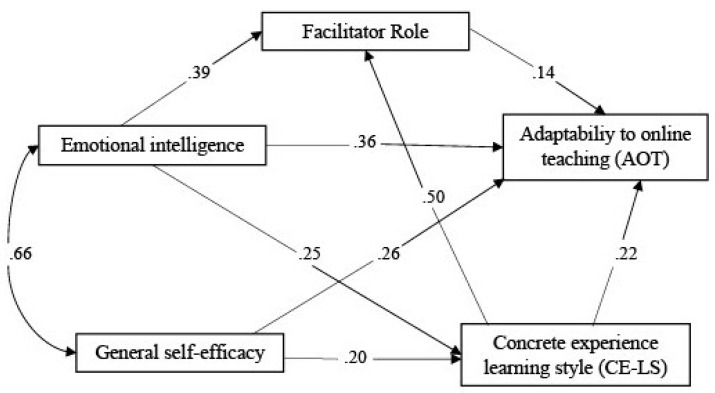
Mediation model of the relationship between EI, general self- efficacy, and AOT.

The strengths of each contributing pathway in the mediation model (as shown in Figure 2) were calculated using standardised path coefficients. The results showed that all paths were significant. More specifically, AOT was predicted by EI (β = 0.36, *p* < 0.001), general self-efficacy (β = 0.26, *p* < 0.001), CE-LM (β = 0.22, *p* < 0.001), and the Facilitator role (β = 0.14, *p* < 0.001). In addition, CE-LM was also predicted by EI (β = 0.25, *p* < 0.001) and general self-efficacy (β = 0.20, *p* < 0.001). Other paths revealed the Facilitator role was predicted by EI (β = 0.39, *p* < 0.001) and CE-LM (β = 0.50, *p* < 0.001).

Testing the impact of EI on AOT revealed that the total indirect effect via both mediators, that is, CE-LM and the Facilitator role, was statistically significant (estimates, 95% bootstrap confidence intervals and bootstrap *p*-values are shown in Table 3). The outcomes evidenced partial mediation because the direct effect of EI on AOT remained statistically significant after controlling for the mediator variables (as shown in Table 3). The null hypotheses of no mediation were rejected for all variance estimators because none of the bootstrap confidence intervals included zero. Therefore, the first two sub-hypotheses were confirmed. The outcomes indicated that specific indirect effects via both mediators had similar values. Additionally, we noted that the serial mediation effect was also significant (as shown in Table 3); thus, the third subhypothesis was validated.

Checking the various effects of general self-efficacy on AOT, we also obtained partial mediation, considering that the direct effect remained statistically significant after controlling for the mediating role of CE-LM (as depicted in Table 4). Thus, the fourth subhypothesis was confirmed.

In addition, we checked gender differences in terms of AOT based on a one-way ANOVA analysis. The results obtained, that is, F (1, 328) = 0.221, *p* = 0.638, proved that no significant gender differences were found in AOT.

## 5. Discussions

This study explores AOT and its associated factors among primary and preschool teachers during the COVID-19 pandemic. The study found that AOT is facilitated by dispositional traits such as general self-efficacy and EI but also by CE-LM and the teacher’s Facilitator role. The major strength of our research is that, although the changes produced in the teaching process during the pandemic were previously explored, the predictors and mediators of AOT analyzed in Kolb’s ELT [14] framework have not been studied to date, as far as we know.

The first key fact from the findings of the study was that AOT is significantly positively associated with dispositional traits such as EI and general self-efficacy. In this sense, although the link between online teaching and EI and the link between EI and adaptability to change have been discussed previously [25,26,28], the relationship between EI and AOT during the COVID-19 pandemic context has not been highlighted. In this sense, our findings showed that the ability of teachers with increased EI to understand and manage emotions both themselves and in others helped them to constructively regulate their behaviors in response to a new and challenging pandemic online education context. Concerning the significant positive correlation between AOT and general self-efficacy, this finding is in line with other studies that have found a positive correlation between teacher self-efficacy and AOT [13,51]. In addition, our findings are complementary to Johnson et al.’s [52] study, which suggested that the lack of physical presence in the classroom affected the teacher’s self-efficacy during the COVID-19 pandemic, more precisely by decreasing it, in the context of online teaching experience [53,54].

Second, the results of the current study showed for the first time in the literature a significant positive correlation between AOT on the one hand and CE-LM and the Facilitator role on the other. One explanation for this finding could be that the Facilitator role is characterised by a warm affirmative style, open to experience, which emphasizes personal relationships and inside-out learning, traits that promote overcoming obstacles and adversities in the instructional-educational process both in classroom and online teaching. According to Kolb [14], teachers who learn in a concrete way prefer the Facilitator role. Moreover, our findings emphasized that entering the learning spiral through the role of facilitator ensures the best adaptability to online learning, the focus being the learner and the meaning instead of the matter or the action.

Third, the present study supplied evidence for the predictive role of EI, general self-efficacy, CE-LM, and the Facilitator role on AOT. Those teachers who have an increased EI, high general self-efficacy, and prefer both the Facilitator role and learning with concrete experience have more chances to adapt to online teaching.

Fourth, the specified multiple mediation model was fit to the data. As shown in the path analysis, CE-LM and the teacher’s Facilitator role had a serial mediating role in the relationship between EI and AOT. Additionally, the relationship between general self-efficacy and AOT was mediated by the CE-LM. This pattern highlights that it takes more than high general self-efficacy and increased EI to be a teacher adapted to online teaching. Indeed, a teacher’s Facilitator role with a strong preference for CE-LM comprises those psychological dimensions that complement and describe a teacher being just an efficacious and empathic teacher vs. being an adaptable teacher in online learning. It is natural that teachers with high EI adapt online to a greater extent than those with a low level because they can understand and manage students’ emotions, even if they are not face to face. Moreover, due to EI, they can self-regulate the frustrating emotions generated by the shortcomings of computer-mediated interaction by the reduced possibility of intervention and help given to students. It seems plausible that efficacious/adapted teachers in online teaching contexts are those with high EI who predominantly use learning strategies based on concrete experience and prefer a Facilitator role, precisely because these characteristics imply (i) creating vivid learning experiences; (ii) stimulating students’ curiosity and cognitive engagement; (iii) practical applicability of knowledge; and (iv) focusing on the teacher-student relationship and on inside-out learning. In addition, our findings pointed out that the relationship between general self-efficacy and AOT was mediated by the CE-LM. In this sense, we can argue the Facilitator style represents the teacher’s emotional intelligence in teaching online action and concrete experiences learning mode because the richness of techniques and learning methods through feeling and watching represents a way to be efficacious in the remote instructional process.

Despite the strengths and contributions of these findings, certain limitations exist. Because the main aim was to assess various predictive relationships and not to establish causal links, future research should consider examining longitudinal and experimental designs to deepen an understanding of the association between AOT and a Facilitator teacher’s role. Second, to ensure that as many participants as possible were recruited for the study, the participants were not randomly selected across the study area. Instead, they were recruited through online and snowballing techniques and, hence, might not be representative of the population.

Future research is needed to expand empirical support for AOT predictors and mediators. More specifically, it is necessary to study the combined influence of facilitators, but also of the negative factors of AOT, such as (i) poor coping strategies with massive stressors encountered in online teaching, (ii) low levels of flexibility and openness, and (iii) lack of spontaneity in adapting to unpredictable and challenging situations.

## 6. Conclusions

The contribution of this research consists of extending the existing body of research on AOT during the COVID-19 pandemic by filling the knowledge gap regarding the mediating paths of the association between general self-efficacy, EI, and AOT. As mentioned above, to our knowledge, the present study is the first to date to investigate the topic of AOT based on Kolb’s ELT [20]. The findings of the current study highlighted that teachers’ online adaptability during the COVID-19 pandemic was influenced by dispositional traits such as EI, general self-efficacy, and teacher’s Facilitator role and CE-LM. This study provides a complementary perspective on previous studies, highlighting how EI, general self-efficacy, and constructs from ELT [14], such as a teacher’s Facilitator role and CE-LM, can lead to a greater AOT among preschool and primary school teachers. All in all, the implications of this study for pedagogical online practice emphasize that, even though most of the studies conducted so far on remote teaching during the COVID-19 pandemic focused on teacher’s technological burnout due to the lack of experience with digital devices and digital teaching [10], psychological traits matter, including EI and general self-efficacy, as well as the teacher’s Facilitator role and CE-LM. In other words, equipping people with the technological skills needed to cope with unexpected changes favors but is insuffient for effective AOT. Beyond the importance of high self-efficacy, the promotion of emotional skills to establish deep interpersonal connections, both physically and online, with the students represents important predictors of successful AOT. In this regard, interactive tutorials that require autonomy, online reading journals, personal stories in a blend of synchronous and asynchronous chat, and discussions with peers and instructors, as well as lectures that focus on interpretations [55], can provide a sense of connection and belonging, even in distance learning. Other implications must be considered concerning training teachers to use concrete experience-learning strategies that can ensure viable solutions for remote teaching.

## Figures and Tables

**Table 1:** Descriptive statistic—mean, SD, skewness, and kurtosis.

Variables	Mean	SD	Skewness		Kurtosis	
			Statistic	Std. Error	Statistic	Std. Error
AOT	37.55	14.81	0.22	0.134	−1.21	0.268
General self-efficacy	31.25	6.09	−0.30	0.134	−0.86	0.268
EI	147.33	23.81	0.36	0.134	−0.36	0.268
Facilitator role	7.05	2.51	0.02	0.134	−1.13	0.268
CE-LM	41.01	12.61	0.28	0.134	−0.82	0.268

**Table 2:** Correlation matrix between research variables.

Research Variables	1	2	3	4	5
AOT	-				
EI	0.64 **	-			
General self-efficacy	0.54 **	0.65 **	-		
CE-LM	0.50 **	0.59 **	0.36 **	-	
Facilitator role	0.56 **	0.38 **	0.35 **	0.67 **	-

** *p* < 0.01.

**Table 3:** Direct, indirect, and total effect of EI on AOT.

Various Effects of EI on AOT	Estimate	Lower	Upper	*p*
EI → Facilitator → AOT	0.06	0.01	0.09	0.001
EI → CE-LM → AOT	0.05	0.01	0.08	0.001
EI → CE-LM → Facilitator → AOT	0.02	0.01	0.03	0.006
Total indirect effect	0.13	0.11	0.17	0.001
Direct effect	0.36	0.35	0.54	0.000
Total effect	0.49	0.38	0.53	0.000

**Table 4:** Direct, indirect, and total effect of general self-efficacy on AOT.

Various Effects of General Self-Efficacy on AOT	Estimate	Lower	Upper	*p*
Indirect effect: General self-efficacy → CE-LM → AOT	0.05	0.01	0.09	0.008
Direct effect: General self-efficacy → AOT	0.26	0.23	0.29	0.001
Total effect	0.31	0.28	0.34	0.001

## Data Availability

The data are available for those who want to see it with justified reasons. Kindly contact the corresponding author.

## Appendix A

**Table A1:** AOT depending on various socio-demographic variables.

Socio-Demographic Variables		Adaptability to Online Teaching (AOT)			
		Low Level	Medium Level	High Level	Total
Gender	Male	14	47	23	84
	Female	47	125	74	246
Environment	Urban	34	114	89	217
	Rural	27	58	28	113
Level of education	Preschool	16	54	38	108
	Primary school	45	118	59	222
